# Supplementary material for: Maternal separation with early weaning impairs neuron-glia integrity: non-invasive evaluation and substructure demonstration
Source: Sci Rep. 2020 Nov 10;10:19440. doi: 10.1038/s41598-020-76640-y (PMC7656452; doi:10.1038/s41598-020-76640-y)

Maternal separation with early weaning impairs neuron-glia integrity: non-invasive evaluation and substructure demonstration

Haiyan Zeng[1,2], Xiaolei Zhang[3], Wenqiang Wang[2], Zhiwei Shen[3], Zhuozhi Dai[3], Zhijia Yu[1], Shuqin Xu[4], Gen Yan[3], Qingjun Huang[1], Renhua Wu[3], Xi Chen[5], & Haiyun Xu[1,4,6*]

[1]The Mental Health Center, Shantou University Medical College, Shantou, China. [2]Xianyue Hospital/Xiamen Mental Health Center, Xiamen, China.

[3]Department of Medical Imaging, The Second Affiliated Hospital, Shantou University Medical College, Shantou, China.

[4]Department of Anatomy, Shantou University Medical College, Shantou China.

[5]McLean Imaging Center, McLean Hospital, Harvard Medical School, Belmont, USA.

[6]The School of Psychiatry, Wenzhou Medical University, Wenzhou, China

Correspondence and requests for materials should be addressed to HX (email: hyxu@stu.edu.cn)

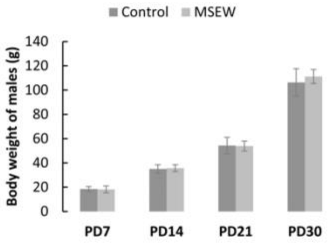

Supplementary Fig. 1. Effects of MSEW on weight gain of rats. The body weight of rats was weighed on PDs 7, 14, 21, and 30, respectively. Data were expressed as mean ± SD. Two-way ANOVA was performed for rats in the two groups with time and treatment as two main factors. One-way ANOVA was performed for rats in each group. Independent t-test was done to compare body weight of rats in the two groups at each time point.

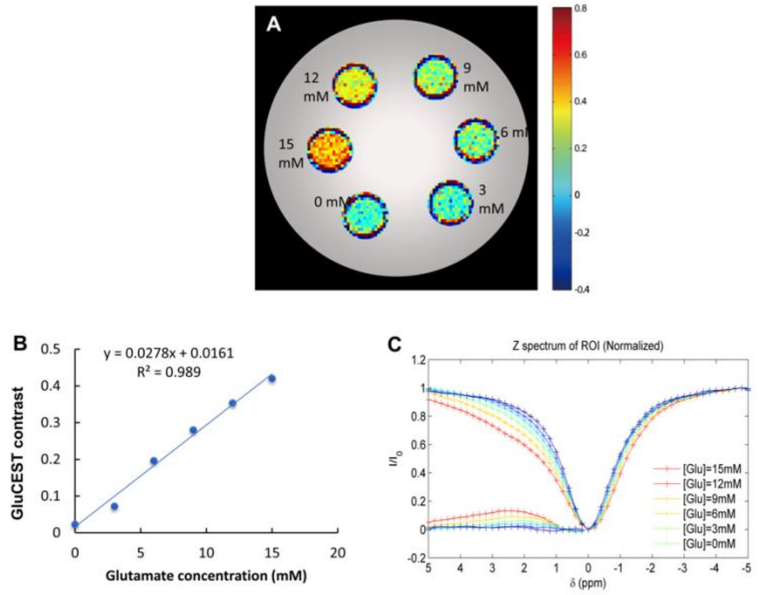

Supplementary Fig. 2. Phantom CEST imaging of Glu solutions in PBS (pH = 7.0). (A) Original CEST images of Glu solutions at various concentrations acquired by applying a saturation continuous wave with a B1 amplitude of 5.9 μT (250 Hz) for 1 sec. (B) Linear correlation between GluCEST contrast and Glu concentration. (C) Corresponding z-spectra and MTRasym curves of the Glu solutions.

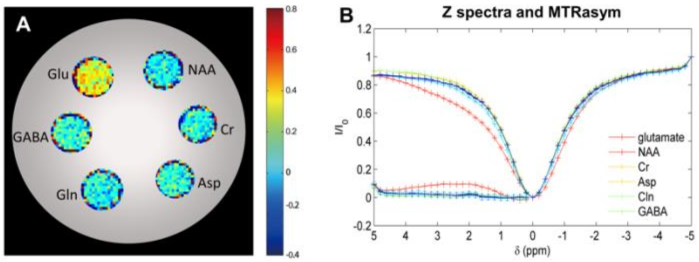

Supplementary Fig. 3. Phantom CEST imaging of some brain metabolites. (A) Original CEST images of the solutions Asp (2 mM), Cr (6 mM), GABA (2 mM), Glu (10 mM), Gln (2 mM), and NAA (10 mM). (B) Corresponding z-spectra and MTRasym curves of the solutions.

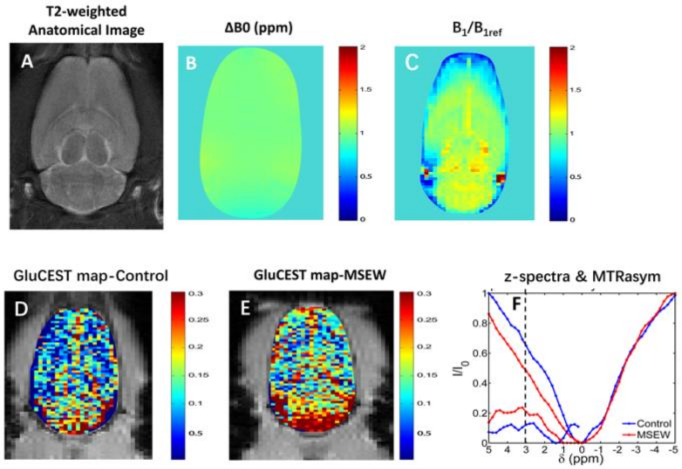

Supplementary Fig. 4. MSEW leads to global GluCEST contrast increase in the rat brain. (A) A T2-weighted anatomic image of the second horizontal slice (thickness = 2 mm) that crosses through the dorsal hippocampus and PFC. (B) A representative B_0 map of the target slice. (C) A representative relative B_1 map of the target slice. (D) The GluCEST map acquired at 3 ppm in the target slice of a rat in the Control group. (E) The GluCEST map acquired at 3 ppm in the target slice of a rat in the MSEW group. (F) Corresponding z-spectra and MTRasym curves acquired in the target slice of the rats.

Original western blot images

Figure 5. Molecular underpinnings of impaired neuron-astrocyte integrity in MSEW rats.

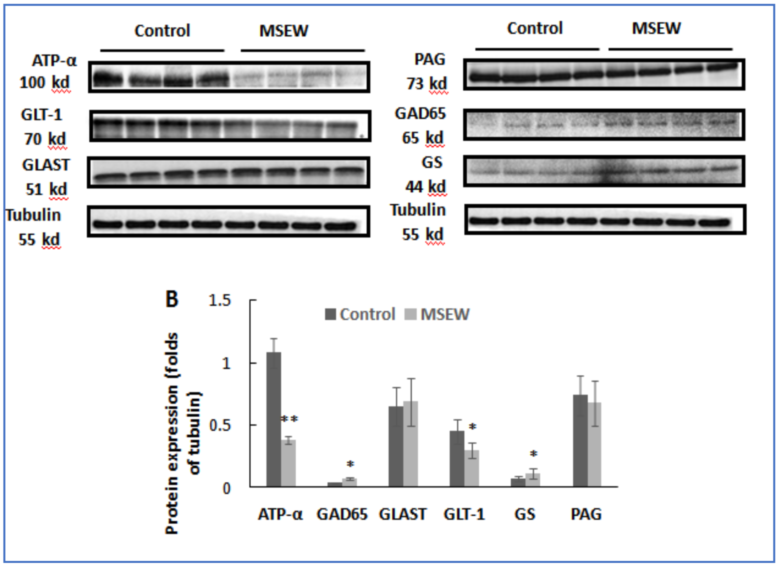

Original Western-blot images of ATP-a, GLT-1, GLAST, and Tubulin in the left column of the upper panel of Figure 5

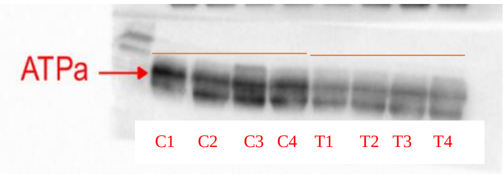

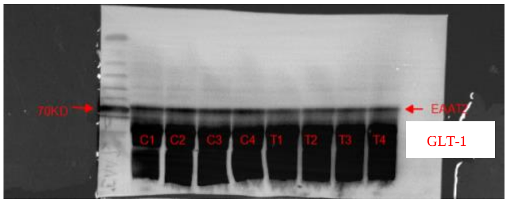

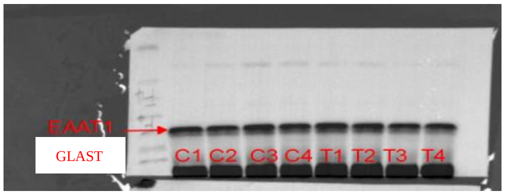

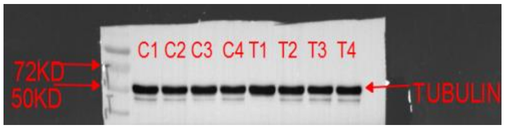

Original Western-blot images of PAG, GLAD65, GS, and Tubulin in the right column of the upper panel of Figure 5

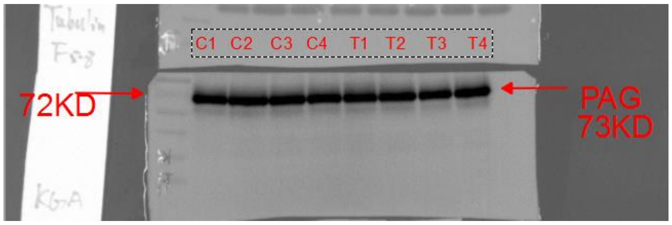

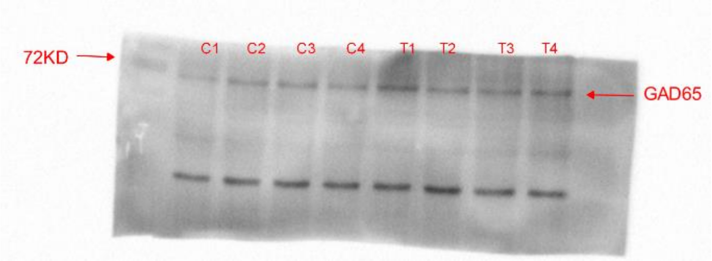

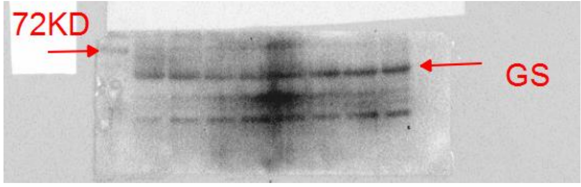

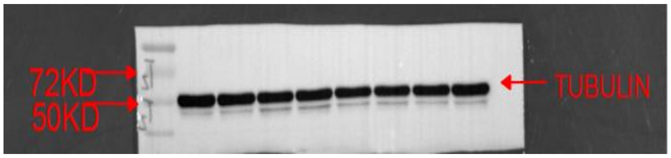

Figure 6. MSEW decreased levels of ASPA and NAT8L in PFC of the rat.

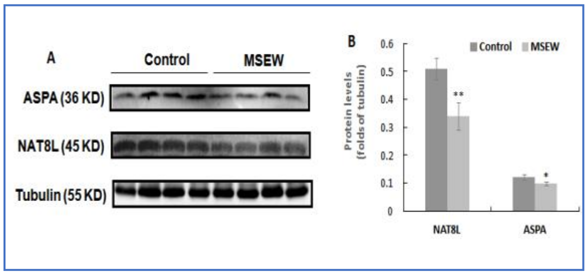

Original Western-blot images of ASPA, NAT8L, and Tubulin in Figure 6

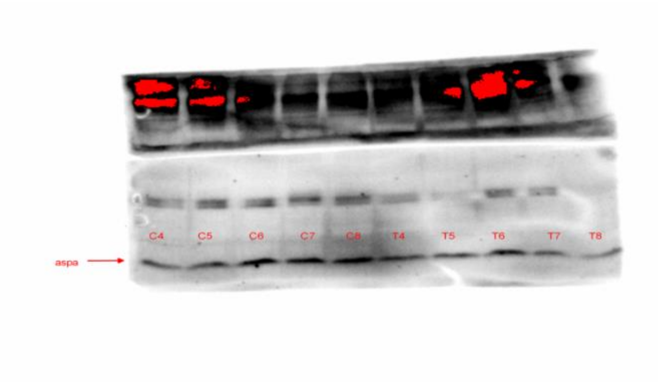

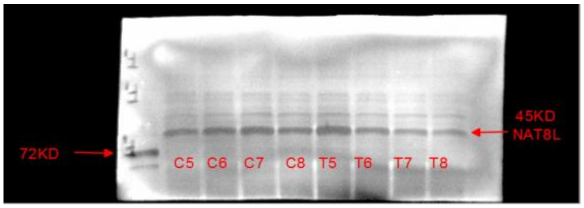

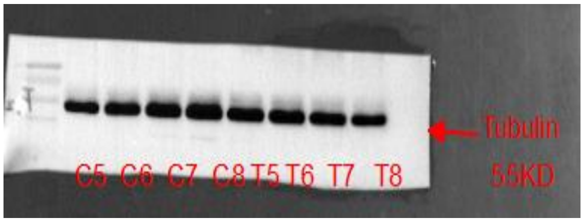

Figure 7. MSEW inhibited OLs maturation and myelination in PFC of rat brain.

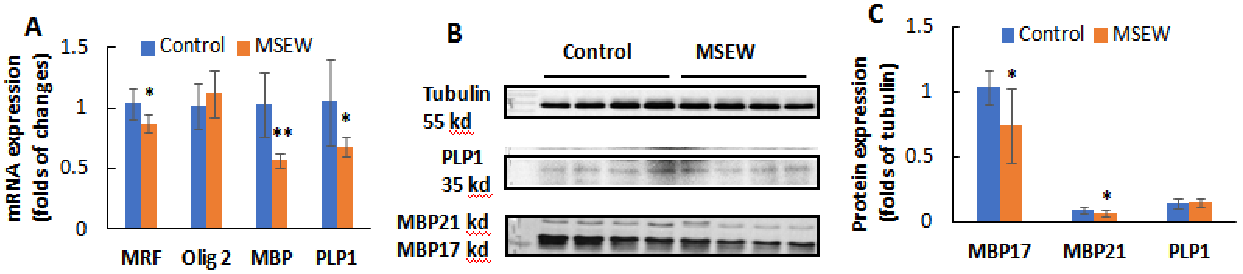

Original Western-blot images of PLP1, MBP21, MBP17, and Tubulin in Figure 7

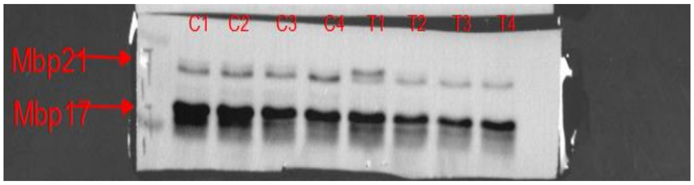

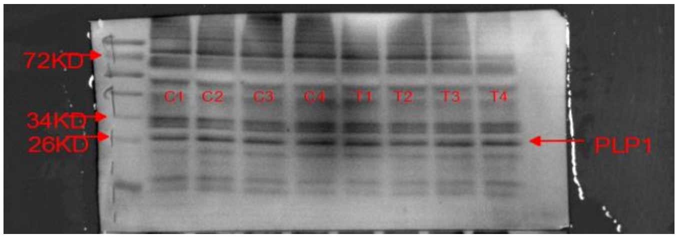

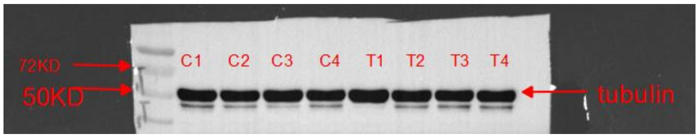

Supplement: Supplementary file 1 — Supplementary information. [file 41598_2020_76640_MOESM1_ESM.pdf]
